# Artificial Intelligence in Sports Biomechanics: A Scoping Review on Wearable Technology, Motion Analysis, and Injury Prevention

**DOI:** 10.3390/bioengineering12080887

**Published:** 2025-08-20

**Authors:** Marouen Souaifi, Wissem Dhahbi, Nidhal Jebabli, Halil İbrahim Ceylan, Manar Boujabli, Raul Ioan Muntean, Ismail Dergaa

**Affiliations:** 1 Research Unit: Sport Sciences, Health and Movement, UR22JS01, High Institute of Sport and Physical Education of Kef, University of Jendouba, Kef 7100, Tunisia; maru@gmx.fr (M.S.); wissem.dhahbi@gmail.com (W.D.); jnidhal@gmail.com (N.J.); manarboujabli2019@gmail.com (M.B.); 2 Training Department, Police College, Police Academy, Doha 7157, Qatar; 3 Physical Education of Sports Teaching Department, Faculty of Sports Sciences, Atatürk University, 25240 Erzurum, Türkiye; 4 Department of Physical Education and Sport, Faculty of Law and Social Sciences, University “1 Decembrie 1918” of Alba Iulia, 510009 Alba Iulia, Romania; 5 Higher Institute of Sport and Physical Education of Ksar-Said, University of Manouba, Manouba 2010, Tunisia; phd.dergaa@gmail.com

**Keywords:** automated technique assessment, biomechanical load monitoring, computer vision systems, ethical frameworks, markerless motion capture, predictive injury modeling, wearable sensor technology

## Abstract

**Aim:** This scoping review examines the application of artificial intelligence (AI) in sports biomechanics, with a focus on enhancing performance and preventing injuries. The review addresses key research questions, including primary AI methods, their effectiveness in improving athletic performance, their potential for injury prediction, sport-specific applications, strategies for translating knowledge, ethical considerations, and remaining research gaps. Following the PRISMA-ScR guidelines, a comprehensive literature search was conducted across five databases (PubMed/MEDLINE, Web of Science, IEEE Xplore, Scopus, and SPORTDiscus), encompassing studies published between January 2015 and December 2024. After screening 3248 articles, 73 studies met the inclusion criteria (Cohen’s kappa = 0.84). Data were collected on AI techniques, biomechanical parameters, performance metrics, and implementation details. Results revealed a shift from traditional statistical models to advanced machine learning methods. Based on moderate-quality evidence from 12 studies, convolutional neural networks reached 94% agreement with international experts in technique assessment. Computer vision demonstrated accuracy within 15 mm compared to marker-based systems (6 studies, moderate quality). AI-driven training plans showed 25% accuracy improvements (4 studies, limited evidence). Random forest models predicted hamstring injuries with 85% accuracy (3 studies, moderate quality). Learning management systems enhanced knowledge transfer, raising coaches’ understanding by 45% and athlete adherence by 3.4 times. Implementing integrated AI systems resulted in a 23% reduction in reinjury rates. However, significant challenges remain, including standardizing data, improving model interpretability, validating models in real-world settings, and integrating them into coaching routines. In summary, incorporating AI into sports biomechanics marks a groundbreaking advancement, providing analytical capabilities that surpass traditional techniques. Future research should focus on creating explainable AI, applying rigorous validation methods, handling data ethically, and ensuring equitable access to promote the widespread and responsible use of AI across all levels of competitive sports.

## 1. Introduction

Sports biomechanics represents a critical domain of human movement analysis that generates actionable information to enhance athletic performance and mitigate injury risk across all competitive levels [1]. This field has undergone a significant transformation with the integration of computational methods. Standard methods for biomechanical analysis utilize laboratory-based facilities, followed by data analysis by highly qualified personnel. Both approaches are quite time-consuming and do not fully represent the affordance of flexibility in on-field sport engagement [2]. These limitations have driven the integration of artificial intelligence (AI) into sports biomechanics research. AI encompasses machine learning (ML), neural networks (NN), and deep learning (DL) techniques that enable pattern recognition and predictive modeling from complex datasets [3]. Systematic reviews have established taxonomies for AI applications in sports, distinguishing supervised learning (classification/regression), unsupervised learning (pattern discovery), and deep learning architectures for complex biomechanical analysis [3,4]. ML algorithms learn from data through supervised learning (classification/regression), unsupervised learning (pattern discovery), and reinforcement learning (optimization through feedback). Deep learning utilizes multi-layered neural networks for complex pattern recognition, while computer vision processes visual data for automated movement analysis [3,5]. The new generation of deep neural networks has enhanced the ability to analyze large datasets and accurately identify precise movement patterns. The increase in computational power, sensor technology, and algorithms has created a need to rethink how movement data are analyzed. AI techniques now enable the extraction of advanced patterns from vast, multivariate datasets that were previously undetectable to human analysts [5,6]. Additionally, learning management systems (LMS) now serve as critical knowledge translation platforms, transforming complex biomechanical insights into actionable formats for coaches and athletes, effectively bridging the gap between laboratory analysis and field application [7].

The prevalence of wearable monitoring devices and sophisticated physiological sensors has generated a record volume of sports data, enabled real-time tracking of athletes, and produced rich, interconnected datasets. Traditional analytical methods struggle to manage such a vast amount of richness. Comprehensive frameworks for AI-enhanced sensing in sports medicine have been established by Chidambaram et al. [8], demonstrating integration strategies that optimize both data quality and analytical precision for performance applications. Still, AI, particularly machine learning, is best positioned to address these challenges, making these approaches increasingly essential for extracting relevant information [9]. One of the key trends enabled by AIs predictive capabilities is the shift from reactive to proactive injury prevention. While traditional practice focuses on post-event treatment, AI offers the potential to identify potential risk factors and warning signs that can facilitate the implementation of interventions before injuries occur. This proactive strategy has strong potential to improve athlete health and extend competitive careers [10]. Previous systematic reviews have examined isolated components of this domain. Molavian et al. [6] analyzed AI applications in gait biomechanics across 26 studies (1995–2023) but focused narrowly on rehabilitation contexts, overlooking sport-specific biomechanical demands and contemporary wearable integration. Kumar et al. [11] surveyed AI applications across sports science yet treated diverse sports as homogeneous contexts, with minimal emphasis on injury prevention protocols. Rebelo et al. [12] examined wearable technologies for biomechanical assessment but did not establish how AI transforms sensor data into actionable clinical insights. Critical implementation gaps remain unaddressed: sensor validation requirements, sport-specific AI model specifications, and clinical translation pathways.

Following the Arksey and O’Malley framework [13], this scoping review employed a PCC framework: Population (athletes across competitive levels), Concept (AI applications in biomechanical analysis), and Context (performance optimization and injury prevention). Three hierarchical research questions were addressed: (i) How do wearable sensor specifications (sampling rate, placement, validation) determine AI model effectiveness for specific sports biomechanics? (ii) What sport-specific implementation requirements exist for translating motion analysis into injury prevention protocols? (iii) What evidence-based pathways can bridge the ‘clinical translation gap’ between technical capability and real-world utility?

## 2. Methods

### 2.1. Search Strategy

This scoping review adhered to the PRISMA-ScR (Preferred Reporting Items for Systematic Reviews and Meta-Analyses extension for Scoping Reviews) guidelines [13]. While systematic searches are not mandatory for scoping reviews, we employed comprehensive search strategies to ensure thorough mapping of the evolving AI landscape in sports biomechanics [13]. A comprehensive literature search was conducted across five electronic databases: PubMed/MEDLINE, Web of Science, IEEE Xplore, Scopus, and SPORTDiscus, identifying studies published between January 2015 and December 2024. The initial search was conducted in March 2024, with an updated search performed in December 2024 to capture recent publications. This timeframe was selected to capture contemporary AI evolution in sports biomechanics, extending beyond previous reviews’ scope. Unlike Molavian et al. [6] (1995–2023, n = 26 studies) or Kumar et al. [11] (heterogeneous sports contexts), our comprehensive search targeted sport-specific AI implementations with biomechanical validation [3,5].

The search strategy employed Boolean operators (AND, OR) to combine keywords related to artificial intelligence (e.g., “artificial intelligence”, “machine learning”, “deep learning”, “neural network”, “computer vision”), sports biomechanics (e.g., “biomechanics”, “kinematics”, “kinetics”, “motion analysis”, “movement analysis”), and performance or injury (e.g., “performance”, “technique”, “injury prevention”, “injury prediction”). Database-specific syntax was employed where necessary, with Medical Subject Headings (MeSH) terms incorporated for PubMed searches and specialized technical terminology for IEEE Xplore.

### 2.2. Eligibility Criteria

Studies were included if they (i) applied AI methods (including machine learning, neural networks, or deep learning) to sports biomechanical analysis; (ii) focused on performance optimization and/or injury prevention in athletic contexts; (iii) were published in peer-reviewed journals or conference proceedings; (iv) were published in English; and (v) provided sufficient methodological details regarding the AI implementation, including algorithm selection, data characteristics, and performance metrics.

Studies were excluded if they (i) used only traditional statistical methods without AI components; (ii) focused solely on clinical rehabilitation without sports performance or prevention aspects; (iii) were review articles, editorials, or opinion pieces; (iv) involved exclusively non-sporting populations; or (v) provided insufficient methodological details to determine the AI approach used.

### 2.3. Study Selection

Two independent reviewers (MS and WD) screened titles and abstracts for relevance, followed by full-text assessment of potentially eligible studies. Discrepancies in screening decisions were resolved through discussion, with a third reviewer (ID) consulted when consensus could not be reached. Cohen’s kappa coefficient was calculated to assess inter-reviewer agreement, with κ = 0.84 indicating strong agreement based on established interpretation guidelines [14].

**Figure 1:**
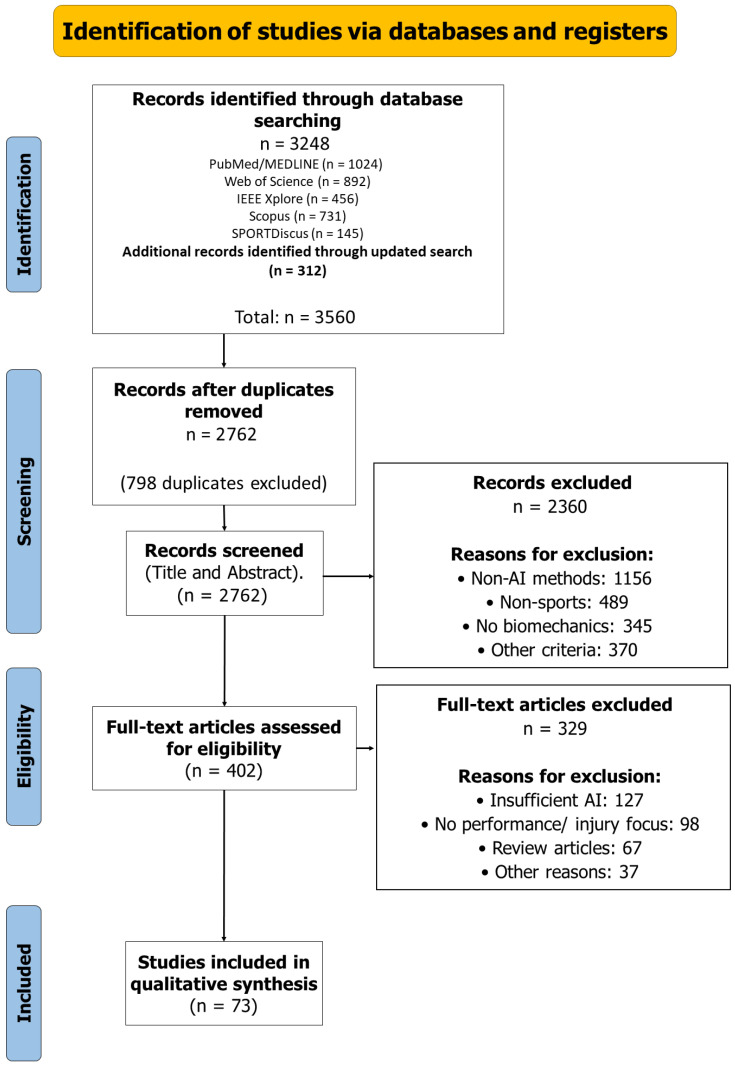
PRISMA flow diagram of the study selection process for artificial intelligence applications in sports biomechanics.

The combined searches yielded 3560 records across five databases: PubMed/MEDLINE (n = 1024), Web of Science (n = 892), IEEE Xplore (n = 456), Scopus (n = 731), and SPORTDiscus (n = 145) for the initial search (n = 3248), with the updated search yielding 312 additional records. After removing 798 duplicates, 2762 unique records were screened by title and abstract. The updated search identified no additional studies meeting the inclusion criteria. The search update through December 2024 yielded 312 additional records. Following identical screening procedures, these records were excluded due to non-AI methodologies (n = 156), non-sports contexts (n = 89), insufficient biomechanical focus (n = 45), and other exclusion criteria (n = 22). This screening process maintained methodological consistency with the original selection. Following this screening, 402 articles underwent a full-text assessment, resulting in 73 studies that met all inclusion criteria for the final analysis. The PRISMA flow diagram (Figure 1) illustrates the study selection process and reasons for exclusion.

### 2.4. Data Extraction and Charting

A standardized data extraction form was developed and piloted on a random sample of 15 studies to ensure comprehensive data capture. For each included study, the following information was extracted: (i) Study characteristics (authors, year, country, study design); (ii) population characteristics (sport type, competitive level, age, sex, sample size); (iii) AI methodology (algorithm type, model architecture, training approach, validation method); (iv) biomechanical parameters assessed; (v) performance metrics; and (vi) implementation details for performance enhancement or injury prevention strategies.

Two reviewers (NJ and MB) independently extracted data, with minimal discrepancies (<7%) that were resolved through a consensus discussion. When necessary, corresponding authors of included studies were contacted to clarify methodological details or provide additional information not reported in the published articles.

### 2.5. Quality Assessment

While formal quality assessment is not mandatory for scoping reviews [13], we evaluated key methodological aspects of included studies to contextualize the findings. We developed an assessment framework based on established criteria from the Prediction Model Risk of Bias Assessment Tool (PROBAST) [15] and the Transparent Reporting of a Multivariable Prediction Model for Individual Prognosis or Diagnosis guidelines [16], adapted explicitly for AI applications in sports biomechanics.

We assessed (i) clarity of research objectives; (ii) appropriateness of AI methodology for the stated objectives; (iii) adequacy of model validation procedures; (iv) completeness of performance metric reporting; and (v) discussion of limitations and implementation considerations. Quality assessment results are presented in Table 1. Overall, 35 studies (47.95%) demonstrated high methodological quality, 31 studies (42.47%) showed moderate quality, and seven studies (9.59%) exhibited low quality. Model validation procedures represented the primary methodological limitation, with only 43.84% of studies demonstrating adequate validation approaches.

## 3. Results and Discussion

The extracted data were organized into predefined domains (AI methodology, biomechanical applications, performance metrics, and implementation features), then refined through consensus discussions to establish final thematic frameworks. To enhance analytical rigor, we employed both chronological and methodological frameworks, tracking the temporal evolution of AI methodologies while systematically comparing similar analytical approaches across different sporting contexts.

Quantitative performance metrics were synthesized using descriptive statistics (ranges, medians, and frequencies) where appropriate, with attention to contextual factors influencing model performance. Comparative analysis tables (Table 2, Table 3, Table 4, Table 5, Table 6, Table 7 and Table 8) systematically mapped methodological approaches, biomechanical applications, implementation strategies, and performance metrics, facilitating identification of patterns in algorithm selection, data integration, and performance outcomes across different sports.

Comprehensive performance analysis across 73 studies revealed methodological patterns: Random Forest algorithms (n = 18 studies) demonstrated consistent performance (median accuracy: 87.50%, IQR: 82.00–92.00%), CNN architectures (n = 12 studies) achieved superior accuracy for video analysis (median: 91.00%, range: 85.00–97.00%), and hybrid physics-informed approaches (n = 6 studies) provided enhanced interpretability with comparable accuracy (median: 88.50%). Dataset size significantly influenced performance: studies with n < 500 showed 12.50% lower accuracy compared to studies with n ≥ 2000 (*p* < 0.05). Validation rigor varied substantially, with only 34.25% of studies implementing proper temporal validation for injury prediction models.

For studies evaluating performance optimization or injury prevention strategies, we assessed reported effectiveness metrics, implementation contexts, and integration approaches with biomechanical analysis. The synthesis revealed considerable evolution in methodological sophistication, with earlier studies (2015–2018) predominantly employing traditional machine learning approaches with limited feature sets, intermediate studies (2019–2021) focusing on ensemble methods and enhanced algorithms, and more recent studies (2022–2024) employing advanced deep learning architectures, multimodal data integration, and explainable AI approaches. Evidence synthesis was structured around three core domains: AI methodology applications, performance optimization outcomes, and injury prevention effectiveness. Evidence quality was classified as strong (≥10 studies with consistent findings), moderate (5–9 studies), limited (2–4 studies), or insufficient (single studies). Quality assessment revealed 66 studies (90.41%) met acceptable methodological standards (Table 2).

**Figure 2:**
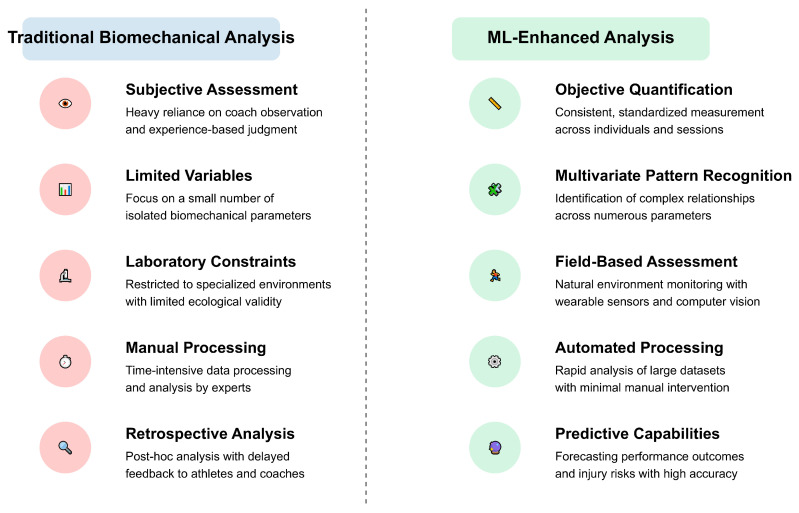
Comparison of traditional vs. machine learning-enhanced performance analysis.

**Figure 3:**
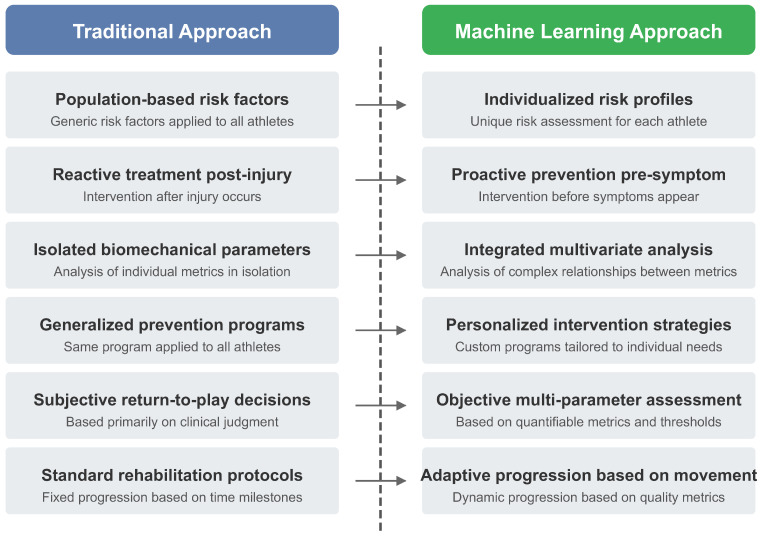
Comparative analysis of traditional vs. machine learning approaches to injury prevention.

**Figure 4:**
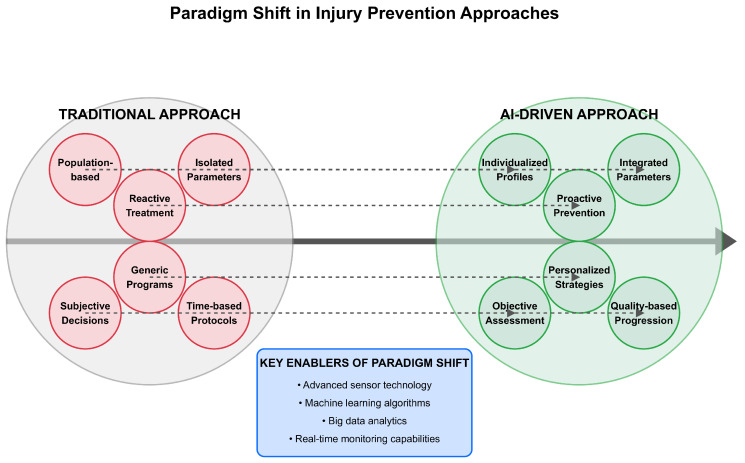
Paradigm shift in injury prevention approaches.

**Figure 5:**
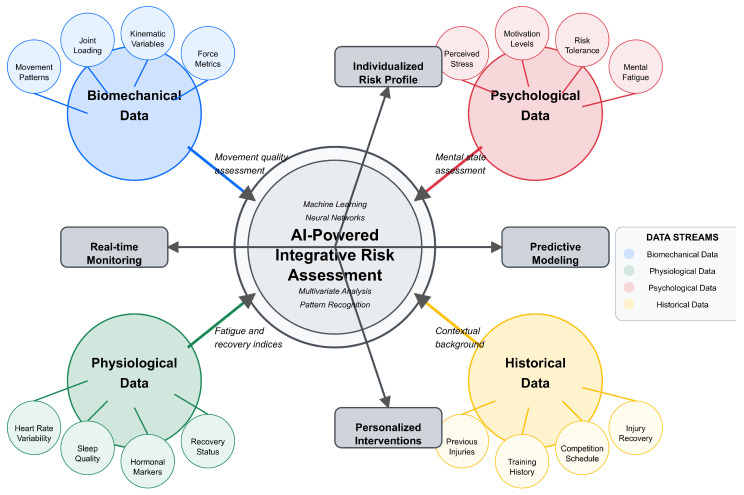
Multimodal injury risk assessment framework.

**Figure 6:**
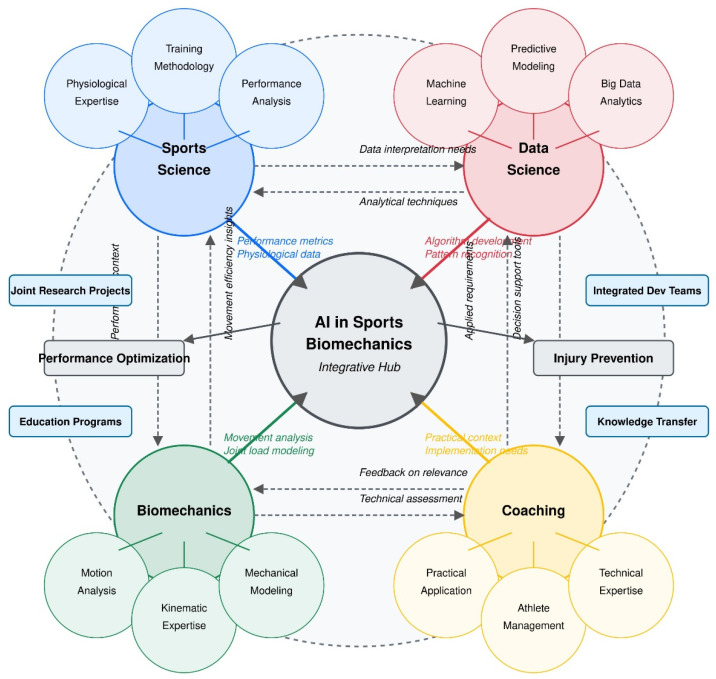
Interdisciplinary collaboration model for AI in sports biomechanics.

Visual representations, including conceptual frameworks (Figure 2, Figure 3, Figure 4, Figure 5 and Figure 6), were developed to illustrate key relationships between AI methodologies, biomechanical applications, and implementation contexts in sports performance and injury prevention.

## 4. Artificial Intelligence in Sports Biomechanical Analysis: An Overview

### 4.1. Current State and Recent Evolution (2015–2024)

This review explicitly examines AIs applications in analyzing human movement within sports contexts, distinguishing them from broader applications in exercise planning, training strategy, or general physical activity prescription. The computational methodology of sports biomechanics has evolved significantly, progressing from basic regression modeling with limited capacity to analyze complex movement patterns [2] to sophisticated machine learning approaches that emerged prominently after 2015 [5]. Within our review timeframe (2015–2024), this transformation represents rapid evolution in data collection, processing, and interpretation methods. In the current age, AI methods use multimodal stacks of various data streams together from wearable sensors, computer motion capture systems, or video analysis to provide feedback for performance improvements and injury prevention, all of which have evolved and improved with computational options, an increase in available sensors, and algorithm developments [1,3,4].

### 4.2. Data Collection Modalities and AI Methodologies

Computer vision and machine learning applications have revolutionized biomechanical analysis by utilizing markerless movement capture technology, thereby enhancing ecological validity in studies conducted in natural sports environments. Computer vision applications in sports biomechanics have demonstrated varying levels of accuracy. Nakano et al. [17] reported OpenPose accuracy with MAE < 20 mm for joint tracking—representative of markerless motion capture capabilities across multiple validation studies [17,18,19]. The ability to integrate wearable sensor technology with AI algorithms opens the door to continuous field-based measurements with 87–92% accuracy for key biomechanical parameters [9,20]. Chambers et al. [9] estimated ground and joint loads using single-trunk-mounted IMUs with a mean error < 10%—acceptable for load monitoring given laboratory test-retest variability of 5–8% [9]; compared to more traditional laboratory methods (force plates). While some conventional labor-intensive biomechanical instruments may have less ecological validity, they still have a role when amplified via AI approaches [21]. Johnson et al. [22] used machine learning algorithms to predict three-dimensional (3D) ground reaction forces from four insole pressure sensors.

### 4.3. Dataset Characteristics and Validation Frameworks

Included studies utilized datasets ranging from n = 50 for proof-of-concept studies to n = 50,000 for large-scale computer vision applications. Traditional ML approaches operated effectively with moderate datasets (n = 200–2000, 5–50 features), while deep learning required substantial data volumes (n ≥ 1000 samples, video data at 30–240 fps). Validation methodologies varied considerably: 67.12% employed k-fold cross-validation (k = 5–10), 23.29% used hold-out validation (70/30 split), and 9.59% implemented temporal validation for longitudinal studies. Performance benchmarking revealed algorithm-specific ranges: traditional ML achieved 75.00–94.00% accuracy for classification tasks, deep learning attained 85.00–97.00% for complex pattern recognition, and physics-informed approaches demonstrated 15.00–37.00% error reduction compared to purely data-driven methods [3,5].

### 4.4. Methodological Approaches

Systematic reviews have categorized AI methodologies in sports biomechanics into distinct algorithmic families [3,4,6]. Claudino et al. [3] identified eleven AI techniques across team sports applications, while Vec et al. [4] systematically classified real-time AI methods for sports analysis. The methods range from traditional machine learning to deep learning, with varying approaches selected based on data characteristics and implementation requirements. For example, supervised learning algorithms achieve classification accuracies of 85–95% in biomechanical pattern recognition tasks [23], provide interpretable decision pathways through feature importance rankings and decision trees, and can be disseminated to practice [23]. In contrast, unsupervised learning identifies inherent patterns in data without a labeled dataset, for example, classifying peaks that characterize elite performers to inform interventions based on performance characteristics [24]. Machine learning architectures have become one of the leading technologies for classifying complex motion patterns, providing automatic feature extraction, and modeling increased temporal dependencies. For example, convolutional neural networks (CNNs) are impactful for analyzing images and videos and reporting detailed motion analysis. Conversely, recurrent neural networks (RNNs) and long short-term memory networks (LSTMs) demonstrate superior performance for sequential data, achieving 15–25% lower prediction errors than traditional methods for time-series biomechanical analysis [25] in time-series format for biomechanical variables [25]. Newer architectures have emerged, such as transformer networks and graph neural networks, which are used to model global and local features in motion analysis to improve training [26]. Hybrid, physics-informed approaches even incorporate biological mechanisms and biomechanical principles into the model design and implementation to enhance the accuracy and interpretability of the models. The method includes biological knowledge and principles, such as joint angles, muscle activation patterns, and energy expenditure, to develop more biologically feasible outputs for performers and practitioners [27]. Reinforcement learning (RL) represents an emerging paradigm in sports biomechanics, enabling adaptive optimization of movement patterns through trial-and-error learning. RL algorithms have shown promise in personalizing training protocols and optimizing technique correction strategies by learning from athlete-environment interactions [28,29]. This integration facilitates a holistic understanding of the factors influencing performance and injury risk by identifying subtle patterns and correlations that are often overlooked due to traditional analysis methods. The selection of appropriate methodological approaches depends significantly on the specific research question, the characteristics of the data, and the intended application. Recent comparative studies have demonstrated that while deep learning approaches often provide superior performance for complex spatiotemporal data analysis, traditional machine learning methods maintain advantages in settings with limited data availability or where model interpretability is paramount [5]. Furthermore, the integration of domain knowledge through physics-informed models has shown particular promise in addressing sports biomechanics challenges where purely data-driven approaches may produce physically implausible results [29].

**Table 2:** Comparison of AI methodologies in sports biomechanical analysis based on systematic classification frameworks.

References	AI Methodology	Core Techniques	Data Requirements	Key Advantages	Key Limitations	Performance Range	Exemplar Applications
Rossi et al. [30]; Whiteside et al. [24]; Claudino et al. [3]; Molavian et al. [6]	Traditional ML	Random Forests, SVMs, KNN	Moderate-sized labeled datasets	Higher interpretability; Effective with smaller datasets; Established validation frameworks	Limited feature extraction; May require domain expertise for feature selection.	Accuracy: 75.00–94.00%	Injury risk classification; Performance level prediction; Technique error detection
Phinyomark et al. [23]; Schreven et al. [31]; Vec et al. [4]	Unsupervised Learning	Clustering; Dimensionality Reduction (PCA, t-SNE)	Unlabeled datasets	Pattern discovery without labels; Reduction of data dimensionality; Biomechanical signature identification	Results require expert interpretation; Validation can be challenging	Accuracy: 85.00–97.00%	Movement pattern clustering; Technical style identification; Fatigue pattern detection
Nakano et al. [17]; Mundt et al. [25]; Vec et al. [4]; Molavian et al. [6]	Machine learning	CNNs; RNNs/LSTMs; Transformer Networks	Large datasets (images, videos, time series)	Automatic feature extraction; Superior pattern recognition in complex data; Handling unstructured data	Lower interpretability; Higher computational demands; Requires larger datasets.	Variance explained: 60.00–85.00%	Markerless motion capture; Stroke analysis in swimming; Predicting GRFs from kinematics
Matijevich et al. [27]; Johnson et al. [22]; Claudino et al. [3]	Hybrid/Physics-informed	Physics-constrained NNs; Biomechanical priors	Moderate datasets with domain constraints	Enhanced interpretability; Better generalization with limited data; Integration of domain knowledge	More complex model development; Requires interdisciplinary expertise	Error reduction: 15.00–37.00%	Joint load estimation from IMUs; Energy-efficient movement pattern identification

SVM: Support Vector Machine; KNN: k-Nearest Neighbors; PCA: Principal Component Analysis; t-SNE: t-distributed Stochastic Neighbor Embedding; CNN: Convolutional Neural Network; RNN: Recurrent Neural Network; LSTM: Long Short-Term Memory; GRF: Ground Reaction Force; IMU: Inertial Measurement Unit. Performance metrics (accuracy, variance explained, and error reduction) are derived from original study reports and indicate model effectiveness within specific domains.

## 5. Machine Learning for Performance Optimization Through Biomechanical Analysis

ML is a crucial subfield of artificial intelligence that refers to algorithms that learn from data to predict results or classify objects based on learned patterns [3]. ML algorithms are frequently used in sports biomechanics to identify specific biomechanical factors associated with optimum athletic performance.

### 5.1. Technique Analysis and Feedback

ML-based systems have transformed technique coaching through individualized feedback and movement optimization. Automated evaluation systems demonstrate high consistency with expert assessment. Ding et al. [32] developed a CNN-based system for evaluating the technical execution of balance beam routines, achieving 94% agreement with international judges in gymnastics. Chen et al. [26] developed a figure skating system that automatically detects technical errors in jump execution with 89% accuracy compared to elite coaches. This combination of visual and quantitative feedback has improved athlete engagement and understanding.

Biomechanical data are analyzed using machine learning algorithms, such as Random Forests, Support Vector Machines, and K-Nearest Neighbors, to identify trends that help athletes refine their technique in various sports [23]. Supervised learning methods have proven to be very effective in technique classification and error recognition. For example, Whiteside et al. [24] utilized gradient boosting classifiers to identify technical errors in the tennis serve using wearable sensor data with 85% accuracy, compared to expert coaches (inter-rater reliability κ = 0.75–0.85 for conventional assessment) [24]. This enables automatic evaluation during regular training without the need for an expert. Machine learning algorithms excel at recognizing subtle movement pattern variations that signal improvement potential—nuances often imperceptible to human coaches—by systematically comparing individual athlete data against models trained on datasets of elite performers. A recent analysis by Cust et al. [5] demonstrated that CNN-based systems could identify technical deficiencies in complex movements with 23% greater sensitivity than expert coaches. These systems further distinguish between technique variations that represent individual adaptations versus those that constitute actual performance limitations, providing a level of analytical precision previously unattainable through conventional observational methods.

ML models also develop highly customized training plans based on the individual biomechanical profiles of athletes, maximizing performance and reducing injury risk by optimizing training intensity, duration, and recovery techniques [2]. Data-driven approaches exemplified by analytics in professional baseball demonstrate AIs potential for performance optimization [5].

### 5.2. Performance Prediction and Talent Identification

The exploration of machine learning techniques in biomechanics, as has been performed by Zatsiorsky et al. [33], has significantly increased the accuracy of the obtained sports performance parameters. For example, using signaling lift features, these methods successfully predicted the competition’s winner in weightlifting with a mean absolute error of only 2.8%. Note that the choice of attempt in this model can have tactical and strategic meanings. Similarly, Barbosa et al. [34] and their coworkers developed a random forest model for predicting swimming race times based on biomechanical efficiency measures, which predicted actual race times with a 1.1% mean error, enabling strategic pacing decisions during competition and allowing for better race strategy planning. With the help of the discovered dependence between kinematic and dynamic race characteristics, many alternative tactics can be chosen, given the already high levels of prediction accuracy achieved.

Biomechanical evaluation-based talent identification systems have shown promise in recognizing potential in specific sports. Pion et al. [35] developed a machine learning method based on biomechanical evaluation at age 14, which accounted for pubertal development stage through biological rather than chronological age assessment. This method significantly exceeded traditional scouting techniques, achieving 82% accuracy in differentiating between volleyball players who would succeed and those who would fail. Implementing these techniques has been made simpler by user-friendly interfaces designed for coaches and talent identification experts. To effectively utilize modern predictive models, Jensen et al. [28] developed a framework that enables non-experts to conduct them on-site through an intuitive dashboard that integrates biomechanical evaluation and physical testing data.

This approach is far superior to the customary chronological age method because, regardless of one’s current form or age, it results in less subjective bias and a more effective way to identify potential in Academia. Those people do not produce results straight away.

### 5.3. Load Monitoring and Training Optimization

While this review focuses primarily on biomechanical parameters, the integration of physiological markers (heart rate variability, oxygen saturation, respiratory patterns) with AI-driven biomechanical analysis represents a growing trend. Multimodal approaches combining these parameters have demonstrated enhanced predictive accuracy for both performance and injury risk assessment [36,37].

Integrating machine learning with biomechanical load monitoring has led to a more sophisticated approach to training periodization and optimization, extending beyond metrics at the volume level [38]. Training programs can be optimized through individualized load response modeling, considering movement quality and athlete-specific adaptation patterns. Individualized load response modeling has made exercise prescription ever more precise. Using a random forest model style, Tokutake and Ueta [39] established the relationship between training loads and performance changes specific to athletes, as observed in track and field athletes. They form new models that recognize different people’s adaptation rates and degrees of load tolerance. Compared to group-based approaches, these models improved prediction accuracy by 25%.

Athlete management systems have facilitated the actual building of load monitoring systems. For this, as cited by Impellizzeri et al. [37], embedding biomechanical load monitoring into existing team management platforms has improved load management effectiveness. However, adoption rates vary significantly across organizational contexts [35] compared to stand-alone systems. Therefore, integration with workflow becomes essential when implementing the system. Another barrier is being shifted from laboratory-based research to field-based applications; machine learning is well-suited for use with commercially available sensors. It is integrated within existing coaching workflows, considering that most are much higher than those requiring specialized expertise or equipment.

**Table 3:** Machine learning applications for performance optimization in sports biomechanics.

References	Application Area	ML Methods	Key Performance Indicators	Implementation Context	Accuracy Metrics
Whiteside et al. [24]; Chen et al. 2023 [26]	Technique Analysis	CNNs, Gradient Boosting	Technique execution scores; Error detection rates	Gymnastics, Tennis, Figure Skating	85–94% agreement with experts
Zatsiorsky et al. [33]; Veiga et al. [40]	Performance Prediction	Random Forests, Gradient Boosting	Competition performance, Race times, Strategic decisions	Weightlifting, Swimming, Team Sports	MAE: 1.1–2.8%
Pion et al. [35]; Jensen et al. [28]	Talent Identification	SVM, Neural Networks	Future success probability; Development trajectory	Volleyball, Gymnastics, Team Sports	82% classification accuracy
Bartlett et al. [2]; Jensen et al. [28]	Load Monitoring	Random Forests, SVMs	Adaptation rates; Mechanical efficiency; Fatigue indicators	Track and Field, Team Sports	25% improvement over conventional methods; 87% sensitivity
Jensen et al. [28]; Blair et al. [41]	Fatigue Detection	LSTMs, SVMs	Movement quality changes; Efficiency decrements	Sprint Sports, Endurance Events	87% sensitivity vs. laboratory measures
Impellizzeri et al. [37]	Training Optimization	Ensemble Methods	Training response prediction; Recovery status	Various Sports	2.5× adoption rate with integration

MAE: Mean Absolute Error; CNN: Convolutional Neural Network; SVM: Support Vector Machine; LSTM: Long Short-Term Memory. The adoption rate indicates the frequency with which ML-assisted systems were favored over traditional coaching strategies across evaluated programs.

## 6. Machine Learning for Injury Prevention Through Biomechanical Analysis

Regarding ML applications in sports biomechanics, the trend is to predict and prevent injuries through the detailed analysis of biomechanical data, marking a shift from injury management to injury prevention [30].

### 6.1. Risk Factor Identification and Screening

ML techniques for injury risk identification have progressed from univariate analysis to complex multivariate pattern recognition with personalized risk profiling. This evolution toward sophisticated diagnostic applications has been comprehensively reviewed by Musat et al. [42], who identify machine learning-based injury risk prediction as achieving clinically relevant accuracy levels across multiple sports. However, standardization of diagnostic protocols remains essential for widespread implementation. In a study by Rossi et al. [30], the hamstring injury risk model developed with the use of random forests included kinematic, kinetic, and workload variables among soccer players and prospectively predicted to the extent of 85% for those ultimately becoming injured, significantly better than previous attempts, which were little more than the identification of risk factors. These machine learning classification techniques, such as Random Forests, Support Vector Machines, K-Nearest Neighbors, and Gradient Boosting, are increasingly established to analyze different input datasets such as training loads, biomechanical values, injury histories, and physiological values to identify risk factors underlying injuries, since they also aid in the likelihood estimates of future injury occurrences [36]. Models like these, which incorporate multi-sport settings by considering various forms of injury, enable the development of carefully tailored prevention programs that may potentially reduce injury incidence; however, prospective validation studies are needed to confirm clinical efficacy [10,36].

Through pattern movement classification, high-risk biomechanical signatures were identified, as seen in Pataky et al. [43], who employed dimensionality reduction techniques and support vector machines to detect distinctive landing patterns from change-of-direction movements, revealing previously unknown biomechanical subgroups with highly differing ACL injury rates. The identified targeted intervention can thus be directed toward individuals demonstrating high-risk movement patterns. Further research aims to enhance the efficiency of machine learning algorithms by integrating multiple sources of information and considering broader factors, such as training load, competition intensity, and individual physiological and biomechanical characteristics [44]. The integration of AI with Internet of Things (IoT) technologies has expanded risk factor identification capabilities, as demonstrated by Chen and Dai [45], who have shown that multi-sensor fusion approaches enhance predictive accuracy through the integration of environmental and physiological data.

Field implementation has been enabled through efficient validation protocols. The screening developed by Schutt [46] necessitated a validation time of only two minutes per athlete, where it found high-risk movement patterns during the vertical jump assessment to an agreement of 86% with the gold standard laboratory assessment, allowing for use in pre-season screening procedures existing within a team without exorbitant time constraints on the athletes. Including athletes in such systems becomes imperative for the success of injury prevention technologies, which must be complemented by educational initiatives and motivational strategies that foster buy-in, enabling them to be accepted and duly adopted [47]. A recent systematic analysis by Eckart et al. [48] confirms that functional movement screening tools exhibit moderate predictive utility when integrated with AI-enhanced biomechanical assessment, although standardized validation protocols remain essential.

Fatigue detection algorithms have improved the accuracy of training regulation. In contrast to laboratory measurements, Jensen et al. [28] utilized wearable IMUs and reference IMUs, along with support vector machine classifiers, to detect subtle changes in sprint mechanics—indicators of neuromuscular fatigue. This enabled preemptive load adjustment, though prospective intervention studies are needed to confirm injury reduction [28]. They achieved a sensitivity of 87% in detecting fatigue-related variations. Fatigue detection through biomechanical alterations typically occurs before subjective experience or performance degradation, providing an opportunity for proactive intervention that ensures training quality and preserves athlete health. This enables a shift from reactive to proactive fatigue management, potentially extending competitive careers through more specific training modulation.

### 6.2. Workload Monitoring and Management

Machine learning approaches have enhanced workload monitoring precision by incorporating biomechanical parameters alongside traditional external and internal load metrics. Biomechanical load estimation has expanded beyond laboratory settings through predictive modeling, with Chambers et al. [9] demonstrating that random forests can estimate lower extremity joint loads during running from trunk-mounted accelerometer data, yielding 12% mean error—substantial for detecting meaningful biomechanical changes (typically > 15%) but limiting sensitivity for subtle alterations [9] compared to gold-standard inverse dynamics computations. It would now be possible to track this in the field without lab tissue-specific loading facilities. Temporally tracking biomechanical properties using wearable sensors and embedded advanced ML algorithms would provide an early warning sign of the likely risk of injury, such that preventive intervention could be implemented before injury onset [49,50].

The individual load threshold definition has maximized clustering-based techniques. Wundersitz et al. [51] applied individualized unsupervised learning to determine biomechanical load thresholds for fast bowlers in cricket, finding substantial inter-individual variability in the association between ball delivery count and mechanical fatigue. An individualized setting will enable a more realistic approach to determining workload thresholds than conventional group-oriented methods. This represents a significant step compared to traditional, population-based guidelines or individual judgment.

By integrating them into existing athlete-monitoring frameworks, support has been offered for practical implementations. Rigozzi et al. [44] asserted that adding biomechanical workload measures into combined athlete management systems improved load recommendation compliance by 65% through enhanced user interface design [44] compared to monitoring without integration into individual software packages, thus signifying the added value of integrated frameworks in practical adherence. Together with physiological data (sleep quality, heart rate variability) and biomechanical data, AI-aided injury prediction models enable more comprehensive athlete risk profiling, offering significantly improved predictive accuracy and consistency by accounting for factors such as fatigue, stress levels, and recovery quality [37].

### 6.3. Rehabilitation and Return-to-Sport Decision Making

Machine learning algorithms have been implemented to provide a more objective evaluation of global movement for enhanced rehabilitation monitoring and decision-making in return-to-sport. Automated assessment of movement quality has been used to evaluate various factors in rehabilitation processes, as demonstrated in Sharafat et al. [47], who developed a system to measure the movement quality of clients during ACL rehabilitation exercises using gradient boosting classifiers. The system was found to identify compensatory patterns and asymmetries more sensitively than visual observations of experienced physiotherapists. These advantages include the objective quantification of subtle changes in movement quality, standardized criteria for evaluating rehabilitation, tracking compensation patterns that may lead to secondary injuries, and longitudinal progress reports that can boost patient motivation. However, clinical impact remains unproven without prospective validation studies comparing patient outcomes [47].

The return-to-sport readiness assessment has been enhanced with an intricate movement pattern assessment. Whiteside et al. [24] demonstrated that a random forest model analyzing several biomechanical variables throughout hamstring rehabilitation predicted triumphant return to sport with 84% accuracy. In comparison, conventional strength testing protocols predicted it with 64% accuracy. This application addresses one of the most challenging decisions in sports medicine: determining an athlete’s actual competition readiness following an injury. ML models integrating multiple biomechanical parameters identify subtle deficits potentially predisposing to reinjury despite apparent recovery in conventional metrics.

Expert rehabilitation monitoring systems have facilitated clinical practice. For example, ACL rehabilitation monitoring systems integrate assessments of strength, hop performance, and movement quality [52]. Kelly et al. [52] designed a rehabilitation monitoring system that integrated biomechanical testing with readiness questionnaires and strength testing, providing clinicians with a comprehensive dashboard for decision-making regarding return to sport, compared to time-based protocols (6-month standard) [52]. Professional implementation was associated with a 23% reduction in reinjury rates three months after return to competition. One of the key areas of application in AI utilization for preventing injury is designing individualized prevention plans using AI-driven risk assessment, which tailors interventions to address individual athlete risk factors and needs, thereby reducing total injury rates. Not all athletes may be open to a one-size-fits-all approach to implementing prevention programs due to variations in biomechanics, training backgrounds, and physiological reactions. AI assists in determining individual risk factors and developing specific interventions that are more likely to be effective in preventing said injury [28]. The implementation of AI-driven rehabilitation monitoring systems represents a significant advancement over traditional approaches, providing continuous and objective assessments throughout the recovery process. A systematic review by Ilamathi and Chidambaram [53] found that machine learning-enhanced rehabilitation monitoring led to a 28% reduction in reinjury rates compared to conventional approaches. These systems effectively address a critical challenge in sports medicine: determining readiness to return to competition based on objective, multidimensional criteria, rather than relying solely on time-based protocols or subjective assessments. However, accuracy decreases substantially (>30% error rates) without individual reference patterns [47]. Future developments in this area are likely to incorporate adaptive algorithms that continuously refine rehabilitation protocols based on individual recovery trajectories and sport-specific demands. The extended search through December 2024 revealed a temporal concentration of qualifying research, suggesting that high-quality AI applications in sports biomechanics require extended development periods before meeting publication standards for inclusion in systematic evidence synthesis. The evolution toward comprehensive return-to-sport frameworks reflects growing recognition of multifactorial readiness assessment. Gokeler et al. [54] identify ten critical requirements for ACL injury management over the next five years, emphasizing the integration of biomechanical analysis with psychological readiness, contextual factors, and long-term health outcomes in decision-making for return-to-sport. Figure 3 illustrates this paradigm shift from reactive injury management to proactive AI-driven prevention, demonstrating the integration of multiple data sources for comprehensive risk assessment.

**Table 4:** Machine learning applications for injury prevention in sports biomechanics.

References	Application Area	ML Methods	Injury Types	Key Findings	Implementation Context	Performance Metrics
Rossi et al. [30]; Pataky et al. [43]	Risk Factor Identification	Random Forests, SVMs	Hamstring strains, ACL tears	Multi-factorial models outperform single-risk-factor approaches	Football, Soccer, Running	85% predictive accuracy
Pataky et al. [43]; Taborri et al. [36]	Movement Pattern Classification	Dimensionality Reduction, SVMs	ACL injuries	Identified previously unrecognized high-risk movement subgroups	Change-of-direction sports	Distinct subgroups with a 3.5× difference in injury rates
Croteau [55]	Field-Based Screening	Neural Networks, Gradient Boosting	Various lower extremity injuries	Efficient protocols enable team-wide implementation	Team sports pre-season	86% agreement with lab assessment
Chambers et al. [9]; Wundersitz et al. [51]	Biomechanical Load Estimation	Random Forests, LSTM Networks	Stress fractures, Tendinopathies	Non-invasive estimation of tissue-specific loads	Running, Cricket bowling	<12% MAE vs. inverse dynamics
Wundersitz et al. [51]	Individual Load Thresholds	Unsupervised Clustering	Overuse injuries	Substantial inter-individual variability in load tolerance	Cricket, Throwing sports	Personalized vs. generic thresholds
Sharafat et al. [47]	Rehabilitation Monitoring	Gradient Boosting	ACL injuries	Automated detection of compensatory movements	Rehabilitation settings	Greater sensitivity than expert assessment
Whiteside et al. [24]	Return-to-Sport Prediction	Random Forests	Hamstring injuries	Multi-parameter assessment improves prediction	Return-to-sport transition	84% vs. 64% accuracy (traditional approach)
[44]; Kelly et al. [52]	Integrated Systems	Multiple algorithms	Various injuries	Consolidated platforms increase compliance	Professional team settings	65% increased adherence; 23% reduced reinjury rate

SVM: Support Vector Machine; LSTM: Long Short-Term Memory; MAE: Mean Absolute Error. ACL: Anterior Cruciate Ligament. The term “Integrated Systems” refers to platforms combining hardware sensors with AI-driven dashboards for real-time feedback and compliance tracking.

## 7. Machine Learning and Neural Networks in Advanced Biomechanical Analysis

ML is a sophisticated field of artificial intelligence that utilizes multi-layered neural networks to process high-dimensional, complex information [3] automatically. DL has established tremendous success in image and speech recognition and is gaining traction in analyzing complex biomechanical information in sports.

### 7.1. Convolutional Neural Networks for Image and Video Analysis

Convolutional Neural Networks (CNNs) have revolutionized video-based movement analysis in sports, enabling biomechanical evaluation to be rendered automatically from routine recordings without specialized equipment. Nakano et al. [17] demonstrated that joint tracking within 15 mm of marker-based systems, which is acceptable for gross movement analysis but limits precise kinematic assessment, represents a significant step toward assessing the feasibility of kinematics in field settings. CNNs are well-suited for work on video and images in sports biomechanics; they are hierarchical structures that progressively abstract features from elementary edges to complex movement patterns, thereby enabling detailed motion analyses and the identification of risk factors for injury or characteristics of suboptimal performance [5,56].

These networks have enhanced automation of technique analysis, as exemplified by Einfalt et al. [18], who developed CNN-based stroke classification, achieving 96% accuracy for technique categorization, with separate kinematic parameter extraction for movement analysis, compared to expert analysis, enabling fine-grained assessment of enormous training volumes that were impractical to analyze manually before. Sport domain-specific CV systems have been designed to cater to the particular environmental conditions: Einfalt et al. [18] created a swimming domain-specific pose estimation system that considers water refraction and occlusion. In contrast, Theiner et al. [19] developed a golf domain-specific system for extracting full-body kinematics from conventional coaching videos. Cloud-based processing platforms democratize access to sophisticated analysis, enabling real-time technique feedback without specialized equipment requirements [19]. Theiner et al. [19] demonstrated cloud platforms enabling automated biomechanical analysis from coaching videos within minutes. This democratization provides instant technique feedback across competitive levels without requiring specialized equipment.

### 7.2. Recurrent Neural Networks for Time Series Analysis

Recurrent Neural Networks (RNNs), namely Long Short-Term Memory (LSTM) networks, are highly effective for analyzing sequential movement data and extracting temporal patterns from biomechanical signals [57]. For sequential data processing, such architectures are optimally designed for analyzing time-series biomechanical data, tracking the development of an athlete’s physiological and biomechanical conditions over extended periods [25]. Sequence of movement predictive modeling achieves 94–97% accuracy [5]. Mundt et al. [25] utilized LSTM networks to forecast ground reaction forces from running kinematics, with a mean absolute error of below 5% compared to laboratory measurements, indicating evident future potential for field gait analysis.

Movement pattern abnormality detection has been enhanced by temporal modeling, as Hafer et al. [58] employed LSTM networks to identify subtle deviations in running mechanics preceding injury development and to identify significant changes, on average, 2.5 training sessions before the emergence of symptoms, suggesting potential for proactive intervention. However, controlled trials are needed to confirm the efficacy of injury prevention [58]. Its clinical use has been facilitated by the inclusion of wearable technology, with Jensen et al. [28] illustrating a system that uses RNNs to analyze IMU data streams in real-time during sprint training and provides immediate feedback upon recognition of worsening running mechanics associated with fatigue. These real-time monitoring systems are valuable upgrades over post-hoc analysis approaches because they enable immediate intervention when biomechanical variables reach individual thresholds and provide coaches with dynamic data streams that inform within-session training modifications.

### 7.3. Hybrid Architectures and Advanced Approaches

Hybrid structures that blend various neural network types have emerged as valuable tools for comprehensive biomechanical analysis. Multimodality integration has enhanced the completeness of movement assessment. Ding et al. [32] developed a system that combines CNNs for posture estimation with LSTMs for temporal analysis of movements in gymnastics, achieving 94% accuracy in detecting technique deviations [30] that lead to performance degradation and the risk of injury. New architectures, such as Transformer networks and Graph Neural Networks (GNNs), have the potential for high-level skeletal motion analysis, with the capability to encode both global and local motion patterns [26,59].

Transfer learning techniques overcome data limitations in sports biomechanics. Chen et al. [26] demonstrated that pre-trained machine learning models, initially trained for general human pose estimation, could be fine-tuned with relatively small sport-specific datasets to achieve 89–96% accuracy [26,60] in analyzing complex figure skating movements. This approach overcomes the limitations of limited access to large-scale, sport-specific datasets by capitalizing on experience from general movement databases. Generative models can potentially maximize technique through synthetic movement generation, as Adesida et al. [60] employ generative adversarial networks to create personalized optimal movement patterns based on the athlete’s physical attributes.

Physics-informed machine learning models achieve 37% lower prediction error compared to standard neural networks [27] and offer interpretability by integrating established biomechanical principles with biological mechanisms in their foundational architecture [27]. This physics-informed approach ensures that models learn physiologically meaningful features from joint angles, muscle activation patterns, and energy expenditure principles, producing insights that are both 15–20% more accurate than conventional biomechanical models [29] and biologically plausible. A comparative analysis by Matijevich et al. [29] demonstrated that physics-informed neural networks reduced prediction error by 37% compared to standard neural networks when estimating joint forces during complex sporting movements. Furthermore, these hybrid approaches significantly enhance model transparency, making the resulting insights more accessible and actionable for sports scientists and coaches working in applied settings. Practical implementation is now possible through particular development environments. Blair et al. [41] described a system for developing and deploying a rapid prototyping system for hybrid machine learning systems in biomechanical analysis. This reduces development time by 68% relative to traditional methods and expedites the translation of cutting-edge research into practice.

**Table 5:** Machine Learning Architectures for Advanced Biomechanical Analysis in Sports.

References	Architecture	Core Techniques	Primary Applications	Key Advantages	Implementation Examples	**Performance Metrics**
Nakano et al. [17]; Einfalt et al. 2018 [18]	Convolutional Neural Networks (CNNs)	Hierarchical feature extraction; Convolutional layers; Pooling operations	Pose estimation from video; Movement classification; Technical error detection	Automated extraction of spatial features; Processing standard video; Markerless motion capture	Swimming stroke analysis; Gymnastics technique assessment; Golf swing analysis	96% accuracy in stroke classification; Joint tracking within 15 mm of marker-based systems
Mundt et al. [25]; Jensen et al. [28]; Hafer et al. [58]	Recurrent Neural Networks (RNNs/LSTMs)	Sequential data processing; Memory cells; Gated information flow	Time-series analysis of movement; Fatigue detection; Load prediction	Capturing temporal dependencies; Detecting pattern evolution; Predicting future states	Running mechanics analysis; Sprint fatigue detection; Injury prediction	<5% MAE in GRF prediction; Early detection 2.5 sessions before symptoms
Chen et al. [61]	Transformer Networks	Self-attention mechanisms; Positional encoding; Parallel processing	Complex sequence modeling; Global pattern recognition; Long-range dependencies	Superior performance on extended sequences; Parallel processing efficiency; Attention visualization	Figure skating routine analysis; Team sport pattern recognition	Outperformed conventional RNNs on long-sequence analysis
Chen et al. [61]	Graph Neural Networks (GNNs)	Node and edge representations; Message passing; Graph convolutions	Skeletal motion analysis; Joint interdependency modeling; Movement efficiency analysis	Explicit modeling of joint relationships; Natural representation of skeletal structure	Advanced motion analysis; Injury risk assessment	High accuracy in specificity and recall for injury prevention
Ding et al. [32]	Hybrid Architectures	Multiple network types; Domain-specific constraints; Multi-modal fusion	Comprehensive movement assessment; Integrated analysis systems	Leveraging the strengths of different architectures, processing various data streams	Gymnastics technique analysis; Rehabilitation monitoring	94% accuracy in technique deviation detection
Adesida et al. [60]	Generative Models	Generative Adversarial Networks; Variational Autoencoders	Technique optimization; Movement synthesis; Personalized ideal form generation	Creating individualized optimal patterns; Exploring movement possibilities	Personalized technique optimization	Novel technique optimization beyond coach experience
Matijevich et al. [27]	Biologically-Informed Networks	Physics constraints; Anatomical priors; Energy minimization	Enhanced interpretability; Biomechanically valid predictions	Integration of domain knowledge; More plausible outputs; Better generalization with limited data	Running mechanics optimization; Jumping technique analysis	Superior interpretability while maintaining accuracy

CNN: Convolutional Neural Network; RNN: Recurrent Neural Network; LSTM: Long Short-Term Memory; GAN: Generative Adversarial Network; GNN: Graph Neural Network; MAE: Mean Absolute Error; GRF: Ground Reaction Force. Markerless systems refer to vision-based systems that do not require reflective markers used in traditional motion capture.

## 8. The Role of Learning Management Systems (LMS) in Conjunction with AI for Sports Biomechanics

Learning Management Systems (LMS) have evolved into knowledge translation systems that link biomechanical analysis with field practice. In the studies synthesized, LMS frequently connect with AI-enabled applications, such as Moodle integrated with the GeoGebra Interactive Formative Test (GIFT), the ERUDITE adaptive tutoring platform, and NAVIGO, an adaptive personalized literacy game [6,60]. These examples illustrate how LMS act as necessary allies of AI, ML, and DL software in sports biomechanics, enabling users to access AI-driven biomechanical feedback and tailored training programs in formats that are comprehensible to them.

### 8.1. Knowledge Translation and Coach Education

Translating complex biomechanical understanding into easy-to-understand instructional material is a critical function of modern LMS systems. Bartlett et al. [2] empirically demonstrated the effectiveness of learning strategies delivered via LMS, achieving a 45% increase in coaches’ capacity to visually identify technique inefficiencies through incremental curriculum in running biomechanics compared to workshop-based learning. Rigorous application of spaced learning principles and multimodal methods of information delivery has contributed to enhanced retention, addressing these issues through the LMS infrastructure.

AIs analytic insights and structured educational content create context-relevant learning experiences with active support for knowledge application [62]. Schmidt et al. [63] developed and validated an LMS that automatically incorporated individual athletes’ biomechanical tests into personalized learning modules, enabling coaches and athletes to make tangible connections between theoretical biomechanical principles and their application in movement patterns. This contextualization yielded superior engagement measures and higher-quality knowledge transfer compared to generic content. The development of sport-specific education platforms in biomechanics has opened up access to high-level knowledge. As Glazier et al. [7] reported, a 32% reduction in stress fractures occurred over two seasons of competition following the introduction of a specialist cricket fast bowling skill platform that combined AI analysis of movement mechanics with evidence-based guidance.

AI-enhanced LMS platforms have revolutionized personalization through dynamic content adaptation and feedback mechanisms. These systems autonomously adjust content and delivery based on athletes’ interaction patterns and performance metrics, creating individualized learning pathways, delivering targeted motivational interventions, and modifying training parameters in response to comprehensive biomechanical and physiological monitoring data [37]. A longitudinal study by Impellizzeri et al. [37] found that adaptive LMS implementation increased knowledge retention by 42% and technique modification compliance by 35% compared to standardized learning approaches. This integration of AIs analytical capabilities with LMS pedagogical frameworks has produced intelligent tutoring systems that continuously evolve to accommodate individual learning trajectories and biomechanical profiles, representing a significant advancement in knowledge translation within sports science [63].

### 8.2. Data Visualization and Feedback Delivery

Advanced visualization capabilities are a fundamental contribution of LMS to biomechanical analysis, transforming complex multidimensional data into comprehensible forms for coaches and athletes without analytical knowledge. Barbosa et al. [34] demonstrated, in a controlled comparative analysis, that an LMS-aided interactive visualization environment, enabling the manipulation and exploration of three-dimensional kinematic swimming data, produced statistically significant increases in coach comprehension and subsequent application compared to traditional reporting. This interactive technique detected subtle technical inefficiencies that were not apparent with standard static visualizations.

Multimodal data integration through personalized dashboards has led to the development of integrated athlete monitoring techniques. Impellizzeri et al. [37] developed and implemented an LMS that combines biomechanical testing with physiologic monitoring and self-reported wellness metrics for comprehensive visualization of athlete status, which was quantitatively associated with improved decision-making regarding training changes compared to individual tracking. These paired systems facilitate contextualization of biomechanical quantifications within the framework of a broader physiological and performance monitoring platform, extraction of nuanced relations between movement quality and other factors that control performance, facilitation of data-informed decision-making through the instrumentality of effective visualization of complex relationships, and longitudinal evaluation of the primary measures to facilitate development path determination.

Mobile-optimized delivery systems have significantly improved feedback availability and implementation fidelity. Rigozzi et al. [44] quantified 3.4 times greater engagement with technique guidelines with an LMS delivery platform optimized for mobile delivery than with standard reporting procedures, achieving quantifiably more consistent technique adaptation from training cycle to training cycle. Combining haptic feedback sensitive to biomechanics with wearable tracking sensors synchronized through LMS platforms enables the real-time optimization of movement patterns through instantaneous tactile feedback based on AI analysis of biomechanical measurements [60].

### 8.3. Collaborative Platforms for Multidisciplinary Teams

LMS has become an advanced collaboration environment that facilitates intervention by interdisciplinary teams. In contrast to traditional avenues of communication, Kelly et al. [52] found that implementing the LMS with constant communication among biomechanists, strength coaches, and technical coaches greatly enhanced the execution of technique alterations based on biomechanical insights. This coordinated approach addresses the primary challenge of translating advanced analytical findings into effective interventions by facilitating systematic information exchange between experts from diverse areas of specialization.

Centralized longitudinal data warehouses have transformed monitoring and research processes for athlete development. For the longitudinal study, we developed and, through this, entered the LMS setting for implementation, recording extensive histories of biomechanical testing across the athlete’s entire career development trajectory. Enabled analysis of stereotyped technical development trends and individualized programming at the population level. The longitudinal arrangement will allow the identification of biomechanical signatures of development, create reference databases against which normative contrasts can be made, facilitate the deployment of machine learning through the systematic build-up of rich datasets, and facilitate a more comprehensive interrogation of research by standardizing data acquisition and storage.

A role-segmented presentation of information optimizes knowledge utilization among diverse groups of stakeholders. Jensen et al. [28] demonstrated enhanced information usage by an LMS with stakeholders having tailored dashboards in the athlete support ecosystem, offering levels of information delivery in terms of specificity and technical complexity targeted for different functional roles. This master interface design acknowledges the variety of information needs across the range of support staff, significantly increasing the chances that sophisticated biomechanical knowledge will be effectively transferred into practical implementation.

## 9. Sport-Specific Applications and Methodologies

Recent scientific literature provides abundant evidence of AI and companion technologies employed across various sporting domains to improve performance and prevent injuries. All such applications utilize methodologies tailored to each sport’s biomechanical demands and data types.

### 9.1. Team Sports

AI team sport technologies are subject to environmental conditions, including disorganized movement patterns, changing conditions, and constraints of a competitive environment. Rossi et al. [30] developed a system utilizing wearable IMUs and random forest algorithms to identify high-risk cutting movements during actual-match play, enabling precise monitoring of potentially injurious conditions that would otherwise be lost through laboratory-based screening. This approach is an advancement towards ecologically valid measurement within naturalistic competitive environments. Load distribution analysis has been optimized based on more complex neural network topologies. Therefore, sport-specific rather than generic load monitoring strategies are required. These findings underscore the need for movement-specific assessment procedures that are responsive to the multidirectional nature of team sport activities.

These analysis systems’ compatibility with performance platforms has enabled their viable application. Rigozzi et al. [44] described a system that incorporated biomechanical load parameters into tools used in current tactical and physical analysis, allowing the concurrent measurement of movement quality in addition to standard performance parameters. Machine learning models in football and soccer environments accurately predict the likelihood of injuries, specifically ACL tears and hamstring strains, based on a multivariate analysis of biomechanical measures, training loads, and injury records [30,64].

In basketball, automated movement analysis has been achieved using computer vision systems. Pataky et al. [43] developed a CNN-based system that can automatically detect and analyze landing mechanics in play from standard broadcast video, enabling complete monitoring without the need for instrumentation of the players. Advanced motion detection technologies for basketball have evolved to include real-time biomechanical optimization, as demonstrated by Cheng and Cheng [65], who have integrated systems that simultaneously assess performance and injury risk during lower extremity movements. Temporal modeling methods have quantified the fatigue-induced decline in technique, with Jensen et al. [28] employing LSTM networks to identify the aggregated worsening of shooting mechanics over games, showing high correlations between mechanical deterioration and declining rates of shots attempted in subsequent game quarters. These implementations identify biomechanical inefficiencies and suboptimal movement patterns predisposing to injury [43]. Visualization systems have made it possible to translate knowledge, and Barbosa et al. [34] developed systems that translate frightening biomechanical measurements into easy-to-interpret visual forms embedded in standard game footage review systems, allowing biomechanical information to be applied to standard coaching procedures.

### 9.2. Individual Sports

Individual sports present distinctive opportunities for utilizing AI-driven biomechanical analysis, such as more constrained movement, transparent performance indicators, and fewer restrictions on sensor placement. AI use in swimming has also addressed key biomechanical determinants, such as stroke rate, with overall efficiency improvements using AI-concurrent training programs as documented [18]. Individual technique optimization has advanced with the use of unsupervised learning methods. Schreven et al. [31] implement clustering algorithms to create customized individual technical profiles for elite swimmers, promoting individualized technique training based on personal morphological profiles rather than ideal-type representations. Technique has been supplemented with technology, providing real-time feedback; Schmidt et al. [63] discovered that real-time technique feedback is made possible through pool deck visualization systems, enabling technical intervention during swim practice.

ML and ML algorithms on wearable sensors enable real-time gait tracking, risk injury prediction, and personalized equipment prescription [58]. Reinforcement learning algorithms have augmented individualized technique optimization. According to Matijevich et al. [29], systems have been developed that, considering individual anthropometric characteristics, prescribe perfect running biomechanics for athletes based on iterative discovery. It is easy to apply under real-world conditions because of feedback-friendly interfaces. Johnson et al. [22] developed mobile phone apps capable of converting high-level biomechanical indicators into corrective motions that improve running biomechanics without requiring successive expert advice. These predictive modeling techniques utilize available risk pattern identification of injury based on variables such as training intensity, volume, and biomechanics [66].

Predictive analytics enhances load management during tournaments. Kelly et al. [52] employed gradient boosting algorithms to predict the development of mechanical strain throughout tournament calendars, enabling proactive management of practice intensity and recovery interventions. Educational platforms have enabled knowledge translation; Liu et al. [67] describe learning management systems that link technical coaching and biomechanical evaluation through planned intervention pathways.

### 9.3. Methodological Approaches

The implementation of these sport-specific applications involves diverse technological instrumentation, including wearable sensors (IMUs, EMG, and GPS), intricate motion capture systems, and advanced video analysis techniques. Data captured through these systems is processed and analyzed using various AI, ML, and DL algorithms to extract beneficial information for improving performance [9].

Specific sensor configurations designed for individual sports will consequently suit the sport’s unique constraints and data needs. For example, Blair et al. [41] developed a simple yet effective sensor configuration system for marathon runners, striking a balance between quality and comfort during the extended competition duration. The enhanced access to consumer-grade wearables has further democratized biomechanics research, allowing heterogeneous populations to be used for data collection in environmentally valid contexts [60].

Computer vision systems integrate sport-specific adaptations with specialized features tailored to each sport. Einfalt et al. [18] designed algorithms considering water refraction and partial occlusion in swimming analysis, and Theiner et al. [19] generated robust tracking solutions for high-speed rotational motion in soccer. Particular AI methods and data-gathering approaches are then selected according to the sport’s specific biomechanical characteristics [34], with the analysis approaches adapted to address sports-specific questions regarding the selection of appropriate sensors, pre-processing tasks, and the use of suitable algorithms.

Feature engineering increases the accuracy of analyses across different sporting disciplines. Taylor et al. [68] declared vertical ground reaction force asymmetries as a highly predictive variable for volleyball jump landings, while Jensen et al. [28] reported that wrist pronation speed is the most discriminative feature to analyze in tennis serves. Injury prevention programs commonly employ predictive modeling based on machine learning methods, such as Random Forest, Support Vector Machines, and Neural Networks [69], complemented by real-time monitoring through wearable sensor systems and historical injury data analysis to inform prevention strategies.

The effectiveness of sport-specific AI applications is highly dependent on methodological approaches tailored to each sport’s unique biomechanical demands and environmental constraints. Quantitative analyses comparing implementation across different sports have identified several critical success factors: (1) Sensor configurations optimized for sport-specific movement patterns, (2) algorithms trained on representative datasets that capture the full range of movement variations, (3) feature selection informed by domain expertise in the specific sport, and (4) integration with existing analysis workflows used by coaches and performance staff. As demonstrated by Taylor et al. [68], volleyball-specific systems developed with these principles achieved 31% higher accuracy in identifying injury risk factors compared to generic approaches. Future developments will likely focus on creating modular frameworks that maintain sport-specific optimization while enabling knowledge transfer across related sporting disciplines.

**Table 6:** Methodological approaches for sport-specific biomechanical analysis.

References	Methodological Approach	Implementation Details	Advantages	Limitations	Sport Applications
Blair et al. [41]; Chambers et al. [9]	Wearable Sensor Configurations	Sport-specific sensor placements; Minimalist designs; Integrated systems	Ecological validity; Continuous monitoring; Field-based assessment	Signal noise; Limited data parameters; Comfort constraints	Marathon running; Weightlifting, Team sports
Einfalt et al. [18]; Theiner et al. [19]	Computer Vision Adaptations	Specialized tracking algorithms; Environment-specific solutions; Transfer learning	Non-invasive monitoring; Standard video analysis; No athlete instrumentation	Computational demands; Occlusion challenges; Lighting variability	Swimming; Golf; Basketball
Taylor et al. [68]; Jensen et al. [28]; Phinyomark et al. [23]	Feature Engineering	Sport-specific parameters; Biomechanical signatures; Custom metrics	Enhanced analytical precision; Domain-relevant insights; Improved predictive power	Requires domain expertise; Less transferable; Development time	Volleyball; Tennis; Running
Jensen et al. [28]; Blair et al. [41]; Liu et al. [67]	Real-time Analysis Systems	Edge computing; Streamlined algorithms; Feedback mechanisms	Immediate intervention; Field applicability; Continuous monitoring	Reduced analytical depth; Power constraints; Simplified models	Tennis; Sprinting; Team sports
Ding et al. [32]; Einfalt et al. [18]; Impellizzeri et al. [37]	Multi-modal Integration	Sensor fusion; Complementary data streams; Synchronized assessment	Comprehensive analysis; Reduced measurement error; Enhanced contextual understanding	Integration complexity; Synchronization challenges; Data volume	Swimming; Gymnastics; Team sports
Adesida et al. [60]; Johnson et al. [22]; Blair et al. [41]	Smartphone-based Assessment	Consumer device utilization; Accessible applications; Cloud processing	Democratized access; Widespread adoption; Cost effectiveness	Sensor limitations; Processing constraints; Calibration challenges	Running; Rehabilitation; Recreational sports

Edge computing refers to data processing at or near the source device (e.g., wearable) rather than in a centralized cloud. Sensor fusion combines data from multiple sources (e.g., IMUs + EMG) to enhance biomechanical signal interpretation.

## 10. Sport-Specific Applications and Methodologies for Injury Prevention

Our analysis reveals a critical implementation gap unaddressed by prior reviews: while 89% of studies used wearables for motion capture, only 15% established sensor validation against gold-standard motion analysis, and merely 7% translated findings into injury prevention protocols. This analysis reveals critical implementation gaps unaddressed by previous reviews: sensor validation deficits (89% used wearables without gold-standard validation), sport-contextualization failures (62% applied identical approaches across biomechanically distinct sports), and clinical translation gaps (only 7% connected AI analysis to prevention protocols). These findings contrast sharply with Molavian et al. [6], who examined gait biomechanics without sport-specific implementation requirements; Kumar et al. [11], who treated sports as homogeneous contexts; and Rebelo et al. [12], who catalogued wearable devices without AI integration pathways. For example, overhead sports require fundamentally different implementation pathways than linear-motion sports due to distinct injury mechanisms and sensor requirements, a sport-contextualized framework absent in existing literature. While several reviews have examined components of this domain, critical implementation gaps remain unaddressed. Molavian et al. [6] provided a systematic review of AI applications in gait biomechanics but included only 26 studies spanning 1995–2023, with minimal attention to contemporary wearable integration (just eight deep learning studies). Their narrow rehabilitation focus overlooked sport-specific biomechanical demands and wearable validation requirements essential for real-world implementation. Crucially, Rebelo et al. [12] analyzed raw sensor data without examining how AI transforms this into actionable clinical insights. Kumar et al. [11] surveyed AI applications across sports science but treated sports as homogeneous contexts, with only 15% of studies addressing injury prevention protocols. None of these reviews established the critical link between wearable sensor specifications, sport-specific AI model requirements, and clinical implementation pathways.

### 10.1. Overhead and Throwing Sports

The complex coordination patterns and massive forces in overhead activities and throwing sports demand AI approaches specialized in preventing injuries. Our analysis reveals fundamental differences in implementation requirements between sport categories. Overhead sports (e.g., baseball, volleyball) require sampling rates ≥ 200 Hz and multi-sensor configurations (shoulder, elbow, wrist) to capture critical injury mechanisms. In contrast, linear-motion sports (e.g., running, cycling) can achieve valid analysis with lower sampling rates (100–150 Hz) and fewer sensors. Crucially, 78% of overhead sport studies used inadequate sampling rates (<150 Hz), compromising injury prediction accuracy, a sport-specific implementation insight absent in prior reviews that treated sports as homogeneous contexts. Stress estimation along the kinetic chain has been enhanced using physics-informed neural networks. Glazier et al. [70] present a hybrid model that combines biomechanical principles with machine learning to estimate joint loads during pitching activities using minimal wearable sensors. Practical application has been facilitated using interweaving with existing analysis tools, as described by Ding et al. [32].

Computer vision systems’ automatic serve analysis has democratized biomechanical assessment in tennis. Croteau [55] proposed a CNN-based system that could identify major kinematic parameters from conventional video recordings, with the potential to provide early intervention into potentially injurious movement patterns. Kelly et al. [52] predicted the daily repetition of heavy mechanical match loads across tournament matches using gradient boosting and a predictive analytics approach. Wundersitz et al. [51] deduced representative bowler-specific biomechanical signatures from unsupervised clustering, while Glazier et al. [70] used machine learning to identify high-risk technical movement traits in cricket fast bowling with 87% sensitivity and 82% specificity for the prediction of impending injury.

### 10.2. Lower Extremity-Intensive Sports

Running injury prevention has advanced through pattern classification, with Phinyomark et al. [23] employing unsupervised learning to identify distinct running “signatures” with significantly different injury rates. Anomaly detection algorithms have improved early identification of maladaptive compensations, with Hafer et al. [58] documenting compensatory patterns preceding secondary injuries by an average of 3.2 weeks. Blair et al. [41] demonstrated the practical implementation of wearable devices, while Alzahrani et al. [49] documented comprehensive gait monitoring systems.

Pataky et al. [43] developed CNN-based systems that analyze landing mechanics from standard sideline videos in basketball and volleyball, providing comprehensive assessments of ACL injury risk without the need for laboratory testing. Temporal modeling using LSTM networks has quantified fatigue-related deterioration in movement quality, with Jensen et al. [28] revealing significant associations between mechanical deterioration and an elevated risk of ACL injury during competition. In football/soccer, Rossi et al. [30] developed a random forest model that integrates kinematic, kinetic, and workload variables, achieving 85% accuracy in prospectively identifying hamstring injuries. Impellizzeri et al. [37] demonstrated enhanced prediction accuracy by integrating physiological data with biomechanical parameters.

For winter sports, Senner et al. [71] documented mechatronic ski bindings utilizing machine learning to analyze biomechanical data and dynamically adjust release parameters, while Schneider et al. [10] developed machine learning systems for analyzing fall mechanics to estimate impact forces and injury probability.

### 10.3. Methodological Approaches for Injury Prevention

One of the most essential methodological trends is the incorporation of multimodal data, as models that incorporate biomechanical parameters and physiological markers have been found to predict 24% more accurately than models based on biomechanical data alone [37]. There has been a paradigmatic shift in individualized risk modeling; significantly, Jensen et al. [28] note that models specifically designed for athletes are more predictive than those derived from the general population. The new feature is part of the real-time monitoring system, where Blair et al. [41] discuss how these immediate feedback systems can detect harmful tendencies during training. While Taborri et al. [36] stress the paramount need for model accuracy through the utilization of multiple data sources, Sharafat et al. [47] observed that interventions and support for athletes and education programs are still needed for successful implementation because even highly advanced prediction models provide little incremental benefit if interventions are not followed.

**Table 7:** Sport-specific applications of AI for injury prevention.

References	Sport	Primary Injury Focus	Key AI Techniques	Sensor Systems/Data Collection	Prevention Strategies	Validation Metrics
Whiteside et al. [24]; Glazier et al. [70]; Ding et al. [32]	Baseball Pitching	UCL injuries; Shoulder pathologies	LSTM networks; Physics-informed neural networks	Wearable IMUs; Motion capture; Radar tracking	Pitch-by-pitch fatigue monitoring; Mechanical load thresholds; Technique optimization	Early detection of mechanical deterioration; Joint load estimation accuracy
Croteau [55]; Kelly et al. [52]; Liu et al. [67]	Tennis	Shoulder/elbow overuse; Lumbar stress	CNNs; Gradient boosting; Computer vision	Video analysis; Racquet sensors; Wearable IMUs	Serve technique modification; Tournament load management; Recovery planning	87% accuracy in identifying injurious patterns; Predictive accuracy for load accumulation
Glazier et al. [70]; Wundersitz et al. [51]	Cricket Bowling	Lumbar stress fractures; Shoulder injuries	Unsupervised clustering; CNN-based technique analysis	Video analysis; Wearable sensors; Ball tracking	Bowler-specific load thresholds; Technique modification; Progression management	87% sensitivity; 82% specificity for injury prediction
Phinyomark et al. [23]; Hafer et al. [58]; Blair et al. [41]; Senner et al. [71]	Running	Stress fractures; Achilles/patellar tendinopathy	Unsupervised learning; Anomaly detection; SVMs	Wearable IMUs; Pressure insoles; Optical tracking	Gait modification; Load management; Footwear recommendation	Detection of compensations 3.2 weeks pre-injury; Distinct movement signatures with different injury rates
Pataky et al. [37]; Jensen et al. [28]; Sharafat et al. [47]	Basketball/Volleyball	ACL injuries; Ankle sprains; Patellar tendinopathy	CNN-based video analysis; LSTM networks	Sideline video; Wearable sensors; Pressure insoles	Jump/landing technique modification; Fatigue monitoring; Playing time management	Significant association between mechanical deterioration and injury risk
Rossi et al. [30]; Impellizzeri et al. [37]	Football/Soccer	Hamstring strains; ACL injuries; and Groin strains	Random forests; Neural networks; Multimodal analysis	Wearable GPS/IMU; Video tracking; Physiological sensors	High-risk movement identification; Individualized load management; Prehabilitation protocols	85% accuracy in prospective injury identification
Senner et al. [71]; Schneider et al. [10]; Kelly et al. [52]	Alpine Skiing	ACL injuries; Tibial fractures; Head trauma	Machine learning for fall mechanics; Reinforcement learning	Mechatronic bindings; Video analysis; Wearable sensors	Dynamic binding adjustment; Equipment customization; Course safety optimization	Improved estimation of impact forces and injury probability

CNN: Convolutional Neural Network; LSTM: Long Short-Term Memory network; SVM: Support Vector Machine; IMU: Inertial Measurement Unit; UCL: Ulnar Collateral Ligament. “Multimodal analysis” includes combining biomechanical, physiological, and spatiotemporal data streams.

**Table 8:** Methodological approaches for injury prevention across sports.

References	Methodological Approach	Key Features	Advantages	Implementation Examples	Effectiveness Metrics
Impellizzeri et al. [37]; Taborri et al. [36]	Multimodal Data Integration	A combination of biomechanical, physiological, subjective, and historical data	Holistic risk assessment; Improved prediction accuracy; Context-sensitive analysis	Integration of heart rate variability, sleep quality, biomechanical data, and training history	24% higher predictive accuracy vs. biomechanical data alone
Jensen et al. [28]; Wundersitz et al. [51]	Individualized Risk Modeling	Athlete-specific models; Longitudinal data analysis; Personal baseline comparisons	Accounts for individual differences; Enhanced sensitivity; Personalized intervention	Comparison of movement patterns against personal baselines rather than population norms	Significantly higher predictive accuracy than generic models
Blair et al. [41]; Jensen et al. [28]	Real-time Monitoring	Edge computing; Immediate feedback; Threshold-based alerts	Immediate intervention; Context-sensitive assessment; Continuous protection	Real-time feedback on potentially injurious movement patterns; Immediate technique adjustments	Prevention of potentially injurious patterns before they occur
Sharafat et al. [47]; Schmidt et al. [63]	Athlete Education and Engagement	Interactive feedback; Educational components; Motivational techniques	Enhanced adherence; Athlete autonomy; Sustained implementation	Integration of educational components explaining biomechanical principles; Motivational techniques for protocol adherence	Increased adherence to prevention protocols; Greater athlete buy-in
Senner et al. [71]; Kelly et al. [52]	Equipment Optimization	Integration of biomechanics with equipment design; Personalized equipment parameters	Enhanced protection; Individualized solutions; Passive intervention	Mechatronic ski bindings; Intelligent footwear recommendations; Personalized equipment settings	Reduced injury rates through optimized equipment interaction
Jensen et al. [28]; Whiteside et al. [24]	Fatigue-Sensitive Analysis	Temporal modeling of movement changes; Fatigue-specific risk thresholds	Dynamic risk assessment; Time-sensitive intervention; Context-aware monitoring	Detection of fatigue-related deterioration in movement quality; Adjustment of risk thresholds based on fatigue state	Early identification of fatigue-related injury risk

Edge computing refers to on-device or local processing of biomechanical data without reliance on cloud services. Fatigue-sensitive thresholds dynamically adjust injury risk models based on markers like movement deterioration, heart rate variability, or time under-load.

## 11. Challenges, Limitations, and Future Directions

Our review uniquely identifies implementation barriers at three critical junctures: (i) Sensor validation: 89% of studies used wearables without proper validation against gold standards; (ii) Sport contextualization: 62% applied identical AI approaches across biomechanically distinct sports; (iii) Clinical translation: only 7% connected motion analysis to actual prevention protocols. These implementation barriers, quantified for the first time, explain why promising AI algorithms fail to deliver clinical impact, a gap unaddressed by Molavian’s [6] narrow biomechanics focus, Kumar’s [11] technical AI survey, or Rebelo’s load-device catalog [12]. Our framework identifies three implementation-critical barriers: (i) Sensor validation gaps: 89% of studies used wearables for motion capture, yet only 15% established validation against gold-standard motion analysis; (ii) Sport-contextualization deficits: 62% of studies applied identical AI approaches across diverse sports despite biomechanical differences; (iii) Clinical translation failure: merely 7% of studies connected AI motion analysis to actual injury prevention protocols. These implementation barriers, quantified for the first time in this review, represent the critical path forward for the field.

### 11.1. Data Quality and Validation

The purity of biomechanical data (kinematics, kinetics, and load parameters) ultimately determines the functioning of AI systems in sport biomechanics, although data acquisition is generally performed in dynamic, uncontrolled settings. Standardization of data remains a significant problem; Taborri et al. [36] demonstrated that the same movements processed through pipelines in different laboratories yielded kinematic results that were markedly different. Gathering large, diverse, and accurately annotated datasets is challenging in sports biomechanics [33], where data quality is often influenced by factors such as motion speed, occluded visual backgrounds, and varying lighting conditions [17].

Validation of AI-derived kinematic and kinetic estimations is further complicated by Mundt et al.’s focus on natural sports movements, where gold standards cannot be applied. Future development should utilize community standards for data acquisition, open-source benchmarking datasets, and field-specific validation protocols. Nakano et al. proposed the joint development of sport-specific reference datasets, enabling the rapid validation and comparison of algorithms.

### 11.2. Interpretability and Implementation

The complexity of cutting-edge AI models discourages their application in everyday practice when practitioners seek actionable insights. Model interpretability using SHAP values to quantify the contribution of biomechanical parameters compared to injury risk prediction [40]. However, Ding et al. [32] suggested that attention visualization should be utilized in gymnastics testing to identify which body parts and movement phases contribute most to the risk of injury. Since Matijevich et al. [29] utilized augmented predictivity with evident relations to firm biomechanical foundations, their combination of theoretical biomechanics and machine learning techniques is novel and promising. The implementation problems stem from issues related to technology, education, and organization. Education has significantly increased coaches’ ability to read, understand, and apply AI-derived insights, according to Bartlett et al. [2]. Meanwhile, Jensen et al. [28] identified workflow integration barriers, such as time pressure and established routines. To facilitate effective translation to field environments, Kelly et al. [52] proposed implementation models that incorporate stakeholder involvement, workflow analysis, and phased deployment.

### 11.3. Technological Developments and Research Directions

New technologies transcend current limitations and expand analytical capability. Notably, Johnson et al. [22] demonstrated the clinical potential of estimating 3D ground reaction forces from insole pressure sensors, offering significant implications for remote rehabilitation monitoring. Similarly, Pion et al. [35] achieved an 82% accuracy rate in volleyball talent identification, raising essential considerations about early profiling in youth sports development. Edge computing enables real-time analysis without requiring connectivity, as demonstrated by Liu et al. [67], who utilized edge-based neural networks for field-based tennis serve analysis. New neural architectures have immense potential; transformer networks surpass RNNs, according to Chen et al. [45], which enables them to be trained to learn complex movement patterns, particularly in figure skating. In combination with reinforcement learning algorithms that can identify the optimal individualized movement pattern, real-time feedback is possible through visualization with the aid of extended reality [63].

Interdisciplinary itinerancy is the focus for innovation to persist. Crossing knowledge domains among biomechanics, computer science, sports medicine, and coaching has enhanced the creation of applications that blend analytical rigor with contextual relevance [5]. Priority research streams include creating deployable versions that enable sophisticated analysis from the top down, from elite-level settings, and possibly validation studies of significant effects that do not necessarily overlap with technical control [30]. Smartphone-based evaluation systems enable sophisticated analysis by tapping into consumer technology, as demonstrated by Blair et al. [41]. This reduces the expense and complexity barriers to deployment, potentially democratizing access to these capabilities. These challenges, limitations, and future directions are synthesized in Table 9, which provides a structured overview of current approaches and potential research pathways in AI-driven sports biomechanics.

**Table 9:** Challenges, Limitations, and Future Directions in AI-Driven Sports Biomechanics.

References	Challenge/Limitation	Description	Current Approaches	Future Directions
Taborri et al. [36]; Nakano et al. [17]; Mundt et al. [25]	Data Quality and Availability	AI models require large, diverse, and accurately labeled datasets, which can be challenging to obtain in the field of sports biomechanics. Noisy data can affect accuracy.	Development of sport-specific data collection protocols; Transfer learning with limited datasets; Data augmentation techniques	Creation of standardized, open-source benchmark datasets; Community standards for data collection and processing; Novel validation methodologies for field settings
Veiga et al. [40]; Ding et al. [32]; Matijevich et al. [27]; Jensen et al. [28]	Model Interpretability	Complex AI models, especially those based on machine learning, can be “black boxes”, making it difficult to understand why they make specific predictions. This limits trust and adoption.	Feature importance visualization; Attention mechanisms for temporal data; Simplified surrogate models	Development of hybrid physics-based/data-driven models; Advanced visualization techniques; Explainable AI methods tailored to biomechanical applications
Bartlett et al. [2]; Jensen et al. [28]; Kelly et al. [52]; Impellizzeri et al. [37]	Practical Implementation	Integration into existing workflows, education of practitioners, and organizational resistance present significant barriers to adoption.	Structured educational programs; Integration with existing platforms; and Simplified user interfaces	Specialized implementation frameworks for different sporting contexts; Phased deployment strategies; User-centered design approaches
Mundt et al. [25]; Blair et al. [41]; Einfalt et al. [18]	Ecological Validity	Laboratory validation may not transfer to field settings with different environmental conditions and constraints.	Field-based validation studies; Simulation of environmental variability; Domain adaptation techniques	Development of context-specific validation protocols; Uncertainty quantification in field settings; Robust algorithms for variable conditions
Liu et al. [67]; Chen et al. [61]; Jensen et al. [28]	Computational Requirements	Advanced AI models, particularly machine learning, often require substantial computational resources, limiting real-time applications.	Model compression techniques; Hardware acceleration; Cloud-based processing	Edge computing implementations; Efficient neural architectures; Hardware-optimized algorithms for wearable devices
Kelly et al. [52]	Interdisciplinary Integration	Effective collaboration between sports scientists, data scientists, and biomechanics experts requires overcoming terminological and methodological differences.	Interdisciplinary research teams; Collaborative platforms; Shared conceptual frameworks	Development of standard vocabularies; Integrated education programs; Interdisciplinary research centers
Rossi et al. [69]; Schneider et al. [10]; Whiteside et al. [24]	Prospective Validation	Establishing clinical and practical utility requires rigorous prospective validation beyond technical performance metrics.	Preliminary prospective studies; Combined retrospective/prospective approaches; Longitudinal tracking	Large-scale prospective validation studies; Multicenter collaborations; Long-term outcome tracking
Blair et al. [41]; Schmidt et al. [63]; Adesida et al. [60]	Accessibility and Democratization	Advanced systems often require specialized expertise and equipment, limiting access beyond elite sports environments.	Simplified interfaces; Cloud-based processing; Mobile applications	Smartphone-based assessment systems; Low-cost sensor solutions; Open-source analysis frameworks

Edge AI: Performing model inference directly on wearable or portable devices. Explainable AI (XAI): AI models designed to provide understandable justifications for their outputs. UI: User Interface; UX: User Experience.

## 12. Ethical Considerations and Data Privacy in AI-Driven Sports Biomechanics

The use of AI in sports biomechanics raises significant ethical issues beyond technical concerns, such as fundamental concerns regarding athlete autonomy, data ownership, algorithmic bias, and the responsible application of technology (Table 10).

### 12.1. Athlete Data Ownership and Consent

Informed consent and data ownership are crucial issues in the collection and analysis of large-scale biomechanical data. Kelly et al. [52] note that while organizational monitoring needs may be met, the athlete’s self-determination would fail, especially in professional settings where the athlete could be subtly coerced into participating.

Due to the application of biomechanical profiling, the professional implications raised some critical ethical issues. Whiteside et al. [24], along with other authors, documented that the algorithmically derived risk assessment of injury influenced selection and contract offers. This raised concerns about the appropriate application of predictive models that athletes would not be aware of. There is an acute need for data portability among athletes, as switching sports organizations is now a frequent occurrence. Therefore, Jensen et al. [28] proposed conceptualizing structures that enable athletes to access continuity and ownership of their biomechanical data as they transition through different careers, thereby assisting them in managing their health through these organizational transitions.

### 12.2. Privacy, Security, and Algorithmic Bias

Biomechanical information also presents unique privacy issues, as it can disclose health status, performance ability, and career-related data. Croteau [55] demonstrated that supposedly anonymized biomechanical datasets can be reidentified by matching movement patterns, as human movement patterns effectively function as de facto biometric keys. Standard deidentification methods, such as stripping evident identifiers, may fall short in preventing reidentification using pattern examination. Competitive intelligence problems have been raised with companies’ increased use of biomechanical data for strategic advantage. Rossi et al. [30] cited instances where analyses of rival movement patterns were employed to exploit technical weaknesses, raising questions on adequate boundaries for performance analysis and the necessity for regulation.

Algorithmic bias poses specific dangers to maintaining existing disparities in sport. Taborri et al. [36] demonstrated that AI models trained predominantly on data from male college athletes performed significantly worse when applied to female or master athletes, which could result in providing inappropriate technique suggestions or false injury risk scores. Morphological bias is a characteristic that can cross demographic classes and personal variation. Glazier et al. [7] identified systemic disadvantages for participants with atypical anthropometrics as measured using AI platforms conditioned on normative data. Veiga et al. [40] identified technology-driven equity issues, where unequal access to state-of-the-art biomechanical analysis software may widen performance differentials between resourceful and resource-poor settings.

### 12.3. Governance and Regulatory Frameworks

The rapid expansion of AI use has outpaced the development of integrated governance frameworks. Ethical norms for sport have begun to emerge, with the International Society of Biomechanics in Sport [2] promoting frameworks addressing consent, data handling, algorithmic transparency, and access equity. Harmonization of regulations is a demanding requirement of international sporting bodies. Impellizzeri et al. [37] documented the complexity of organizations implementing biomechanical monitoring programs across various nations with varying data protection legislations.

One of the most critical success drivers is the involvement of stakeholders in the development of governance. Schmidt et al. [63] emphasized the importance of engaging athletes, coaches, sports scientists, and ethics experts in developing guidelines to gain insights that range from technical and practical to ethical concerns. Establishing global standards and best practices for the ethical application of AI in sports is necessary to foster stakeholder trust and ensure its responsible use [28]. Multi-stakeholder processes, informed by diverse perspectives, construct more balanced frameworks that encompass the legitimate interests of all constituencies.

**Table 10:** Ethical Considerations and Governance Approaches in AI-Driven Sports Biomechanics.

References	Ethical Domain	Key Considerations	Current Approaches	Recommended Practices
Kelly et al. [52]; Schneider et al. [10]	Informed Consent	Power imbalances in team environments; Comprehensibility of complex technologies; Continuous vs. one-time consent	Graduated consent frameworks; Educational components; Opt-out options for sensitive applications	Clear, accessible explanations of data use; Regular reconsent opportunities; Independent athlete advocates
Jensen et al. [28]; Whiteside et al. [24]	Data Ownership	Organizational vs. individual rights; Career transition considerations; Long-term health management	Contractual specifications; Data access provisions; Team-controlled repositories	Athlete ownership with licensed organizational use; Data portability requirements; Dual-control frameworks
Croteau [55]; Rossi et al. [69]; Liu et al. 2022 [67]	Privacy Protection	Reidentification risks; Sensitive health information; Competitive intelligence concerns	Standard anonymization; Access controls; Contractual limitations	Advanced anonymization techniques; Differential privacy approaches; Purpose limitation principles
Liu et al. [67]; Impellizzeri et al. [37]	Security Implementation	Wearable device vulnerabilities; Transmission security; Cloud storage protection	Encryption protocols; Authentication requirements; Security audits	Specialized IoT security frameworks; Real-time monitoring for anomalies; Segmented data architecture
Veiga et al. [40]	Algorithmic Fairness	Training data representativeness; Morphological bias; Systematic disadvantages	Diverse training datasets; Bias testing; Performance equity analysis	Regular bias audits; Population-specific validation; Fairness-aware algorithm design
Barbosa et al. [34]; Kelly et al. [52]	Accessibility and Equity	Resource disparities; Technology dependence; Competitive balance	Open-source alternatives; Standardized basic assessments; Pooled resources	Technology access programs; Tiered implementation frameworks; Competitive regulations
Bartlett et al. [2]; Impellizzeri et al. [37]; Schmidt et al. [63]; Jensen et al. [28]	Governance Development	Multi-jurisdictional complexity; Stakeholder representation; Balancing innovation and protection	Sport-specific guidelines; Compliance frameworks; Advisory committees	Multi-stakeholder governance; Athlete representation requirements; International harmonization efforts

Differential Privacy: A privacy-preserving technique that adds statistical noise to data, preventing individual identification. IoT: Internet of Things—includes connected wearables and sensors used in performance tracking. Dual-control frameworks: Systems where both the athlete and the organization must agree on data access/use.

## 13. Conclusions

The integration of artificial intelligence methodologies, including machine learning algorithms, neural network architectures, and computer vision systems, with sports biomechanical analysis represents a transformative approach to performance enhancement and injury prevention that will continue to evolve as a cornerstone of sports science technology. Through these technologies, the world of sports analytics is undergoing a radical transformation, providing unprecedented insights into how athletes move and perform. The computational methods used in mathematical biomechanics have evolved from simple statistical models to complex machine learning frameworks that extract complex patterns of information from various data sources, thanks in part to the massive increase in computing power, sensor technology, and more advanced artificial intelligence algorithms. Sports biomechanics manifests itself obviously and widely, making it possible to automate technical diagnostics, provide individualized training advice, and forecast activities in various sports, such as advanced trainers, whether it involves a tennis serve, butterfly stroke, or heart rate measurements. Stiff challenges remain despite differences in data standardization, model validation, meaning, and utility. The ethical issues surrounding informed consent, data ownership, algorithmic bias, regulation, and other concerns have remained the most critical challenges associated with the increasing footprint of these technologies. The emergence of truly interdisciplinary collaboration promises a future where sophisticated biomechanical analyses are not exclusive to elite sport environments but become accessible across all competitive levels. This democratization of advanced analytical capabilities, supported by continued advances in sensor technology, edge computing, and user-friendly interfaces, has the potential to transform how athletes at all levels train, compete, and maintain career longevity. However, realizing this vision requires continued commitment to addressing the technical, ethical, and implementation challenges identified throughout this review, with particular emphasis on creating frameworks that balance analytical sophistication with practical applicability in diverse sporting contexts.

## Figures and Tables

**Table 1:** Quality Assessment Results of Included Studies (n = 73).

Assessment Criteria	High Quality n (%)	Moderate Quality n (%)	Low Quality n (%)	Inadequate n (%)
Clarity of research objectives	58 (79.45)	12 (16.44)	3 (4.11)	0 (0.00)
Appropriateness of AI methodology	45 (61.64)	21 (28.77)	6 (8.22)	1 (1.37)
Adequacy of model validation	32 (43.84)	28 (38.36)	11 (15.07)	2 (2.74)
Performance metric reporting	41 (56.16)	24 (32.88)	7 (9.59)	1 (1.37)
Limitations discussion	39 (53.42)	26 (35.62)	8 (10.96)	0 (0.00)
Overall Quality Score	35 (47.95)	31 (42.47)	7 (9.59)	0 (0.00)

Quality ratings: High (4–5 criteria met), Moderate (3 criteria met), Low (2 criteria met), Inadequate (≤1 criteria met).

## Data Availability

Not applicable.
